# Oral administration of *Bifidobacterium breve* improves anti-angiogenic drugs-derived oral mucosal wound healing impairment via upregulation of interleukin-10

**DOI:** 10.1038/s41368-023-00263-y

**Published:** 2023-12-11

**Authors:** Qingxiang Li, Yuke Li, Qiao Qiao, Ning Zhao, Yuanning Yang, Lin Wang, Yifei Wang, Chuanbin Guo, Yuxing Guo

**Affiliations:** Department of Oral and Maxillofacial Surgery, Peking University School and Hospital of Stomatology & National Clinical Research Center for Oral Diseases & National Engineering Laboratory for Digital and Material Technology of Stomatology & Beijing Key Laboratory of Digital Stomatology, Peking University School and Hospital of Stomatology, Beijing, China

**Keywords:** Mucositis, Oral manifestations

## Abstract

Recent studies have suggested that long-term application of anti-angiogenic drugs may impair oral mucosal wound healing. This study investigated the effect of sunitinib on oral mucosal healing impairment in mice and the therapeutic potential of *Bifidobacterium breve* (*B. breve*). A mouse hard palate mucosal defect model was used to investigate the influence of sunitinib and/or zoledronate on wound healing. The volume and density of the bone under the mucosal defect were assessed by micro-computed tomography (micro-CT). Inflammatory factors were detected by protein microarray analysis and enzyme-linked immunosorbent assay (ELISA). The senescence and biological functions were tested in oral mucosal stem cells (OMSCs) treated with sunitinib. Ligated loop experiments were used to investigate the effect of oral *B. breve*. Neutralizing antibody for interleukin-10 (IL-10) was used to prove the critical role of IL-10 in the pro-healing process derived from *B. breve*. Results showed that sunitinib caused oral mucosal wound healing impairment in mice. In vitro, sunitinib induced cellular senescence in OMSCs and affected biological functions such as proliferation, migration, and differentiation. Oral administration of *B. breve* reduced oral mucosal inflammation and promoted wound healing via intestinal dendritic cells (DCs)-derived IL-10. IL-10 reversed cellular senescence caused by sunitinib in OMSCs, and IL-10 neutralizing antibody blocked the ameliorative effect of *B. breve* on oral mucosal wound healing under sunitinib treatment conditions. In conclusion, sunitinib induces cellular senescence in OMSCs and causes oral mucosal wound healing impairment and oral administration of *B. breve* could improve wound healing impairment via intestinal DCs-derived IL-10.

## Introduction

Anti-angiogenic therapy is one of the oldest types of cancer treatment. It abolishes the nutrient and oxygen supply to the tumor cells by decreasing the vascular network. Common anti-angiogenic drugs include small-molecule tyrosine kinase inhibitors (TKIs, e.g., sunitinib and cabozantinib) and monoclonal antibodies (e.g., bevacizumab and ramucirumab) that target angiogenesis by inhibiting vascular endothelial growth factor (VEGF) signaling pathways.^1^ Initially, it was believed that this type of therapy is associated with minimal side effects; yet, recent studies suggested systemic inhibition of VEGF may lead to severe complications, including hypertension, thromboembolism, impaired wound healing, and medication-related necrosis of the jaw (MRONJ).^1–3^ Thus, searching for ways to reduce unexpected toxicities caused by anti-angiogenic drugs is required.

The combination of anti-angiogenic drugs and anti-resorptive drugs is a routine medication scheme in cancer patients with bone metastases. However, recent studies have suggested that this therapy may lead to side effects, including MRONJ.^4–6^ One of the most common strategies for treating MRONJ is to completely remove the necrotic bone through surgery and complete soft tissue coverage in MRONJ surgery.^7,8^ Although surgery can lead to good treatment effects in MRONJ, some patients still suffer oral mucosal wound unhealing after the necrotic bone is completely removed. Our clinical observation found that almost all patients receiving anti-resorptive drugs could have a good prognosis. However, applying anti-angiogenic drugs could significantly decrease the wound-healing rate after the operation.^9^ Moreover, an increased incidence of wound healing/bleeding complications, including wound dehiscence (re-opening), was observed in patients who underwent major surgery while receiving VEGF inhibitors-based therapy compared with those receiving chemotherapy alone (13.3% vs. 3.4%).^10^ This indicated that anti-angiogenic drugs might be the major factor in oral mucosal wound healing impairment. Once MRONJ patients develop oral mucosal wound healing complications, the primary tumor treatment may fail due to inevitable adjustments, such as medication treatment breaks, reduced dosing, and drug cessation.

Wound healing is a cascading biological process divided into four distinct but overlapping phases: hemostasis and coagulation, inflammation, cell proliferation, tissue remodeling and maturation. Oral mucosal wounds heal rapidly and rarely scar compared to skin.^11^ Experimental observations indicated that oral mucosal stem cells (OMSCs) have an important role during this process. Researchers have pointed out that excessive inflammatory response may significantly delay the normal wound healing process, while OMSCs can negatively regulate the local inflammatory response of oral mucosal injury, thus affecting wound healing.^12,13^

Several clinical studies have shown that oral mucositis often occurs in patients taking anti-angiogenic drugs, and the incidence rate ranges from 20% to 35.2%.^14–16^ TKIs can also induce cellular senescence in different kinds of cells, such as renal cell carcinoma cells and human umbilical vein endothelial cells.^17,18^ Senescence-associated secretory phenotype (SASP) expression is an important phenotype associated with senescent cells wherein those cells secrete high levels of pro-inflammatory factors.^17^ All this evidence suggests that anti-angiogenic drugs may induce OMSCs senescence, exacerbate local inflammation in the oral mucosal wound, and hinder the healing process.

*Bifidobacteria* is one of the important probiotics in the human body. It widely colonizes the intestinal tract and has a role in biological barriers, enhancing immune function and exerting an anti-tumor role.^19^ Recent studies have demonstrated that *Bifidobacteria* can have a therapeutic role in various inflammatory diseases, suggesting its potential effect in regulating the inflammation response of the host.^20,21^ In the previous study, we found that *Bifidobacterium breve* (*B. breve*) could interact with intestinal dendritic cells (DCs),^19^ which are an important endogenous cellular source of interleukin-10 (IL-10), a well-known anti-inflammatory factor.^22^

A combination of anti-angiogenic and anti-resorptive drugs (e.g., zoledronate and denosumab) is often recommended for cancer patients with bone metastases.^23^ In this study, we investigated the effect of sunitinib and/or zoledronate on oral mucosal healing impairment in mice and the therapeutic potential of *B. breve* on oral mucosal healing impairment caused by sunitinib. We mainly focused on OMSCs senescence, local inflammatory response, and DCs-derived IL-10.

## Results

### Sunitinib drugs impair the healing of the oral mucosal wound

**Fig. 1:**
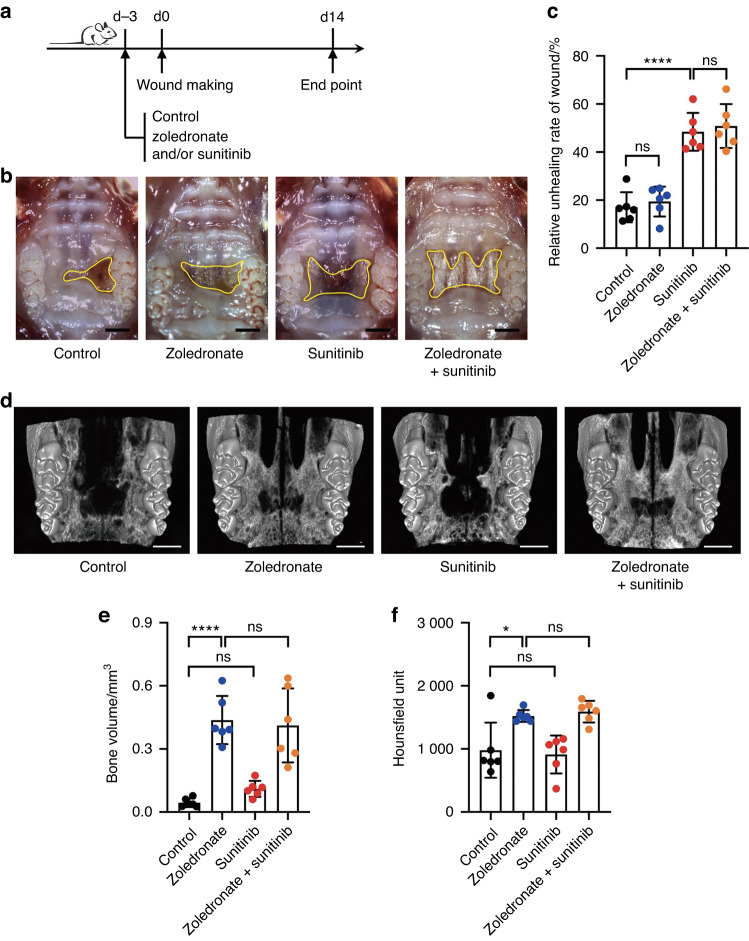
Anti-angiogenic drug sunitinib causes oral mucosal wound healing impairment in mice**. a** Schematic diagram of the experimental protocol. **b** Macroscopic observation shows mucosal wound healing of the palate in mice after different treatments (scale bar: 1 mm). **c** Quantitative analysis of the relative wound area 14 days post-surgery. **d** Micro-CT three-dimensional reconstruction displays bone of palate in mice. Scale bar: 1 mm. **e**, **f** Statistical diagram shows the bone volume (mm^3^) and bone mineral density (Hounsfield unit) of palate bone under the mucosal defect. Each dot represents one mouse in two independent experiments of six mice per group. The data were analyzed with one-way ANOVA and presented as mean ± SD (*P < 0.05; ***P < 0.001; ****P < 0.000 1; ns not significant)

To judge the effect of an anti-angiogenic drug (sunitinib) and an anti-resorptive drug (zoledronate) in the healing process of oral mucosal wounds, we established a mouse model bearing a defect on the hard palate mucosa. After 3 days of drug administration, an oral palate mucosal defect was established, after which the drugs were given for another 2 weeks (Fig. 1a). Sunitinib alone delayed wound healing. The relative wound area in the sunitinib group and zoledronate + sunitinib group was about 2.5 times larger than that in the zoledronate group and control group (*p* < 0.001, Fig. 1b, c).

Given that anti-resorptive drugs impact bone metabolism, we also analyzed the bone of the palate below the wound. Our results showed that the bone volume and bone density of the mice treated with zoledronate (zoledronate group and zoledronate + sunitinib group) increased significantly compared to the sunitinib and control groups (*p* < 0.05, Fig. 1d–f). Thus, these data suggested that oral mucosal healing impairment may be directly related to the anti-angiogenic drug.

### Sunitinib exacerbates local inflammation of the oral mucosal wound

**Fig. 2:**
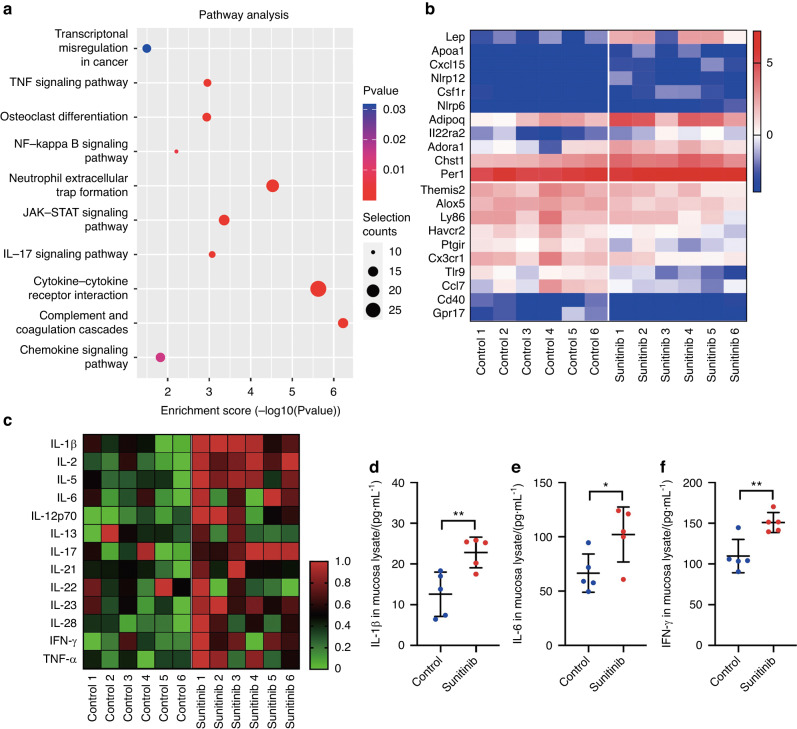
Anti-angiogenic drug sunitinib exacerbates inflammation in oral mucosal wounds. **a** KEGG enrichment analysis for DEGs between the oral mucosa specimen of the mice from the control group and the sunitinib group. **b** Heatmap for DEGs filtered with the condition: |log2(fold change)| > 1. **c** Heatmap for inflammatory factors detected by antibody array. **d**–**f** The expression level of IL-1β, IL-6, and IFN-γ in the mucosa detected by ELISA. The data was analyzed with *t* test and presented as mean ± SD (*P < 0.05; **P < 0.01)

Considering that inflammatory response is an important influence factor of wound healing, we collected the oral mucosa specimen of the mice from the control group and sunitinib group to conduct the genetic and inflammatory pathway sequencing analysis. KEGG enrichment analysis showed that DEGs between the control group and sunitinib group were enriched in the TNF signaling pathway, NF-kappa B signaling pathway, JAK-STAT signaling pathway, and IL-17 signaling pathway, which were closely related to an inflammatory response (Fig. 2a). Specifically, the mainly changed inflammatory genes of the oral mucosa included *NLRP12*, *NLRP6*, *CXCL15*, *IL22RA2*, *CX3CR1*, *TLR9*, *CCL7*, *CD40*, *GPR17*, and similar (Fig. 2b). Based on these, we further detected the expression of major inflammatory factors at the protein level by an inflammatory antibody array. As shown in Fig. 2c, the vast majority of the pro-inflammatory factors (11/13) increased significantly after sunitinib treatment.

Next, the enhanced inflammation level was verified via ELISA test of 3 pro-inflammatory factors (IL-1β, IL-6, IFN-γ, Fig. 2d–f). These results indicated that sunitinib could exacerbate local inflammation of oral mucosal wounds in mice.

### Sunitinib induces senescence in OMSCs

**Fig. 3:**
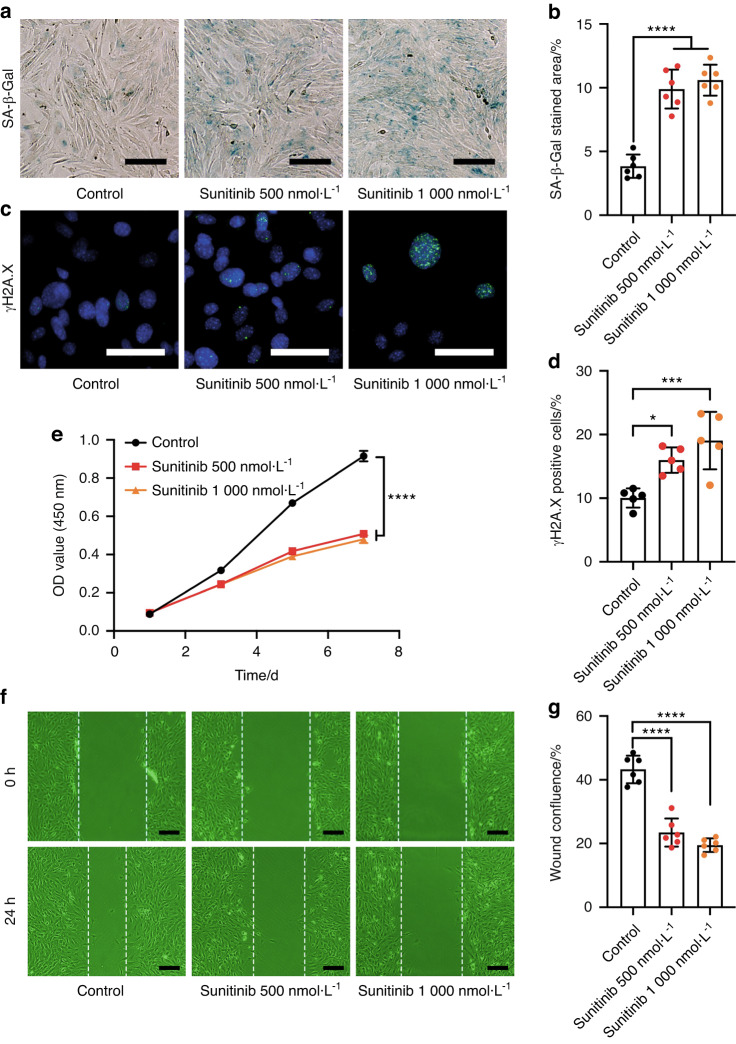
Sunitinib induces senescence in OMSCs. **a** SA-β-Gal staining of OMSCs for examination of cellular senescence (scale bars: 50 μm). **b** Quantitative analysis of the SA-β-Gal positive cells. **c** The expression of γH2A.X in OMSCs detected by immunofluorescence staining assay (scale bar: 100 μm). **d** Quantitative analysis of the γH2A.X positive cells. **e** OMSCs proliferation examined by CCK-8 assay. **f** OMSCs migration examined by wound healing assays (scale bars: 50 μm). **g** Quantitative analysis of the wound confluence after sunitinib treatment for 24 h. Each dot represents one random field. Statistics were two-way ANOVA for the cell-proliferation curve and one-way ANOVA to compare three independent groups. All data were presented as mean ± SD (*P < 0.05; ***P < 0.001; ****P < 0.000 1)

Healing of oral mucosal wounds needs the participation of multiple cells, of which the OSMCs have an important role in different stages, especially the regulation of inflammatory response.^24^ Considering the exacerbation of the local inflammatory response in oral mucosal wounds, we isolated OMSCs from the hard palate mucosa of mice to detect sunitinib’s effect in vitro. These cells were identified as CD44^+^CD105^+^CD34^-^CD45^-^ by flow cytometry (Fig. S1). After adding sunitinib (500 nmol·L^−1^ and 1 000 nmol·L^−1^), the proportion of SA-β-Gal-positive cells in OMSCs increased (Fig. 3a, b). One of the markers for cellular senescence, γH2A.X, is a sensitive molecular marker of DNA damage and repair.^25^ There were more γH2A.X-positive cells in the sunitinib group than in the control group (Fig. 3c, d), which suggested the damage of DNA. Subsequently, sunitinib reduced OMSCs proliferation and migration capabilities (Fig. 3e–g).

In addition, we investigated the influence of sunitinib on the differentiation ability of OMSCs. Results showed that sunitinib significantly inhibited the osteogenesis and adipogenic differentiation abilities of OMSCs (Fig. S2). Furthermore, we detected the mRNA expression levels of SASP (*Il1b*, *Cxcl15*, and *Tnfa*) in OMSCs^17^ and found increased *Il1b*, *Cxcl15*, and *Tnfa* levels after sunitinib (Fig. S3). These data suggested that sunitinib could induce senescence in OMSCs and affect their biological functions in vitro.

### Oral administration of *B. breve* improves oral mucosal wound healing impairment caused by sunitinib

**Fig. 4:**
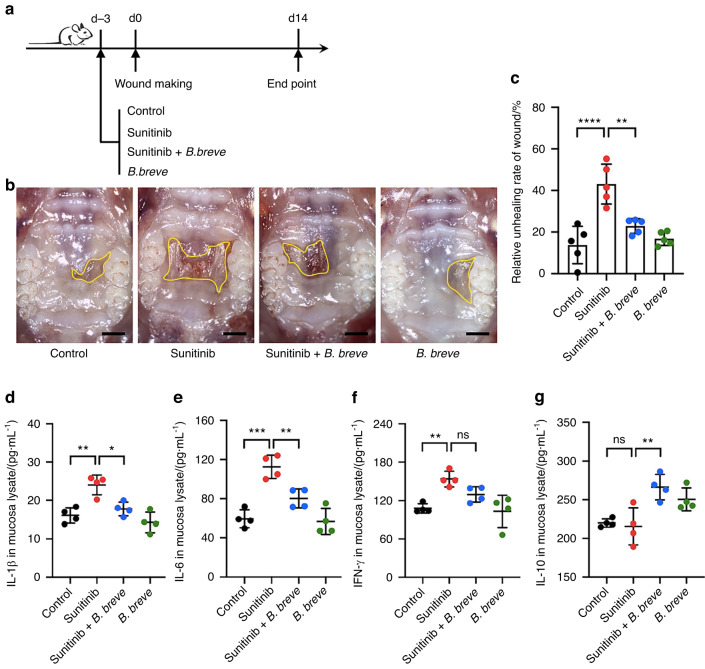
Oral administration of *B. breve* meliorates wound healing impairment caused by sunitinib. **a** Schematic diagram of the experimental protocol. **b** Macroscopic observation shows that mucosal wound healing of the palate in mice after different treatments (Scale bar: 1 mm). **c** Quantitative analysis of the relative wound area 14 days post different treatments. Each dot represents one mouse in two independent experiments of five mice per group. **d**–**g** The expression level of IL-1β, IL-6, IFN-γ, and IL-10 in the mucosa detected by ELISA. The data were analyzed with one-way ANOVA and presented as mean ± SD (*P < 0.05; **P < 0.01; ***P < 0.001; ****P < 0.000 1; ns not significant)

Most cancer patients cannot discontinue anti-angiogenic drugs due to the risk of tumor progression; thus, searching for novel methods to solve oral mucosal healing impairment is urgently required. Herein, we attempted to use *B. breve*, a kind of probiotic, to promote the healing of oral mucosal in mice treated with sunitinib. Experimental results showed that oral administration of *B. breve* could significantly reverse the oral mucosal wound healing impairment caused by sunitinib (Fig. 4a–c). Next, we used the ELISA test to evaluate the abundance of inflammatory factors in the oral mucosa. It was found that *B. breve* could reduce the expression level of IL-1β, IL-6, and IFN-γ in the sunitinib-treated mice (Fig. 4d–f), which indicated a decrease in the inflammatory response. Meanwhile, we noticed that the abundance of IL-10 in the oral mucosa was increased in the mice taking *B. breve* (Fig. 4g). IL-10 possesses an anti-inflammatory function, yet, more testing is required to confirm whether there is a connection between IL-10 and *B. breve*.

### *B. breve* primes secretion of IL-10 from intestinal DCs

**Fig. 5:**
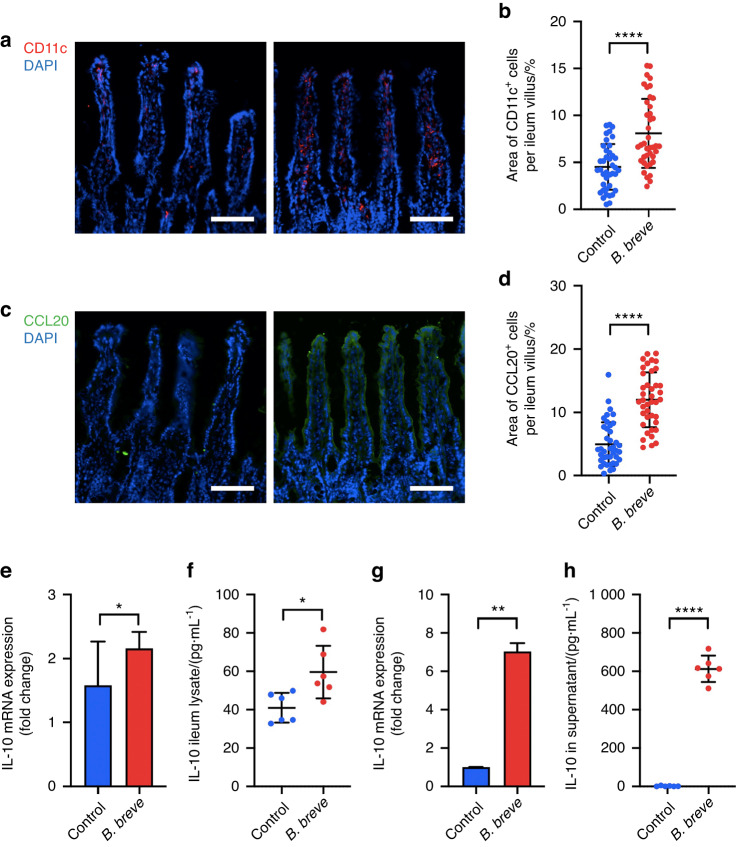
*B. breve* primes intestinal DCs recruitment and IL-10 secretion. **a**, **b** Immunostaining of CD11c (red) and DAPI (blue) of the terminal ileum cryosections. The quantitative analysis and the number of positive cells presented in scatter plots. **c**, **d** Immunostaining of CCL20 (green) and DAPI (blue), the terminal ileum cryosections, and the quantitative analysis presented in a scatter plot. Each dot represents one ileum villus. **e** Relative IL-10 mRNA expression level of the terminal ileum evaluated by qRT-PCR. **f** The expression level of IL-10 in the terminal ileum detected by ELISA. Each dot represents one mouse in two independent experiments of six mice per group. **g** Relative IL-10 mRNA expression level of BMDCs evaluated by qRT-PCR. **h** IL-10 level in the culture supernatant from BMDCs after 24 h of stimulation, as detected by ELISA. Each dot represents one sample in two independent experiments. All these data were analyzed with a *t* test and presented as mean ± SD (scale bar: 100 μm; *P < 0.05; **P < 0.01; ****P < 0.000 1)

An in situ ligated intestine loop model was established to explore the effect caused by oral administration of *B. breve*. Immunofluorescence results showed that *B. breve* recruited more CD11c^+^ DCs migrating into the ileum villus (Fig. 5a, b). In addition, CCL20, the chemokine of immature DCs, was increased in the intestinal epithelium of *B. breve*-treated mice correspondingly (Fig. 5c, d).

Then, we used qRT-PCR and ELISA to detect the expression of IL-10 in the terminal ileum. Our results demonstrated that the treatment of *B. breve* could significantly up-regulate the expression of IL-10 (Fig. 5e, f). In order to prove that DCs were the source of IL-10, we induced and cultured bone-marrow-derived dendritic cells (BMDCs) in vitro. Under the stimulation of *B. breve*, BMDCs expressed and secreted more IL-10 (Fig. 5g, h). Based on the above finding, *B. breve* could recruit intestinal DCs and promote the expression and secretion of IL-10.

### IL-10 improves sunitinib-induced senescence in OMSCs

**Fig. 6:**
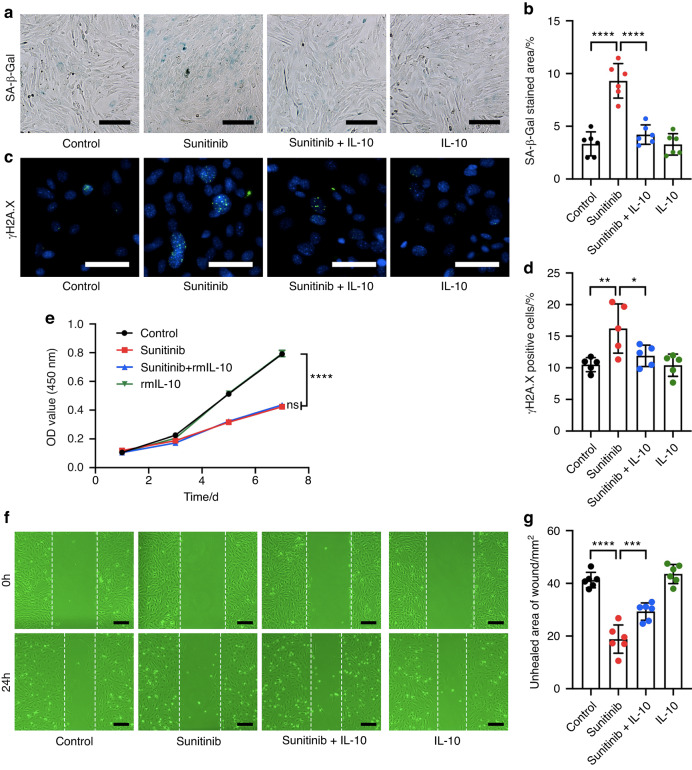
IL-10 improves sunitinib-induced senescence in OMSCs. **a** SA-β-Gal staining of OMSCs for examination of the cellular senescence (scale bars: 50 μm). **b** Quantitative analysis of the SA-β-Gal positive cells. **c** The expression of γH2A.X in OMSCs detected by immunofluorescence staining assay (scale bar: 100 μm). **d** Quantitative analysis of the γH2A.X positive cells. **e** OMSCs proliferation examined by CCK-8 assay. **f** OMSCs migration examined by wound healing assays (scale bars: 50 μm). **g** Quantitative analysis of the wound confluence after treatment for 24 h. Each dot represents one random field. Statistics were two-way ANOVA for the cell-proliferation curve and one-way ANOVA to compare four independent groups. All data were presented as mean ± SD (*P < 0.05; **P < 0.01; ***P < 0.001; ****P < 0.000 1; ns not significant)

Since higher expression of IL-10 was observed in the oral mucosa, we next focused on the influence of IL-10 on OMSCs. With the presence of IL-10, sunitinib-induced senescence of OMSCs was obviously reduced. Specifically, the proportion of SA-β-Gal-positive cells and γH2A.X-positive cells in the sunitinib + IL-10 group was less than that in the sunitinib group (*P* < 0.05, Fig. 6a–d). Furthermore, we detected once more the proliferation and migration capabilities of OMSCs. Compared with sunitinib-treated cells, the addition of IL-10 did not enhance OMSCs’ proliferation capability (Fig. 6e), but the migration capability was partially rescued by IL-10 addition (Fig. 6f, g). The above results suggested that increased IL-10 could largely improve sunitinib-caused inhibition effects on OMSCs.

### *B. breve*-resulted wound healing improvement depended on IL-10

**Fig. 7:**
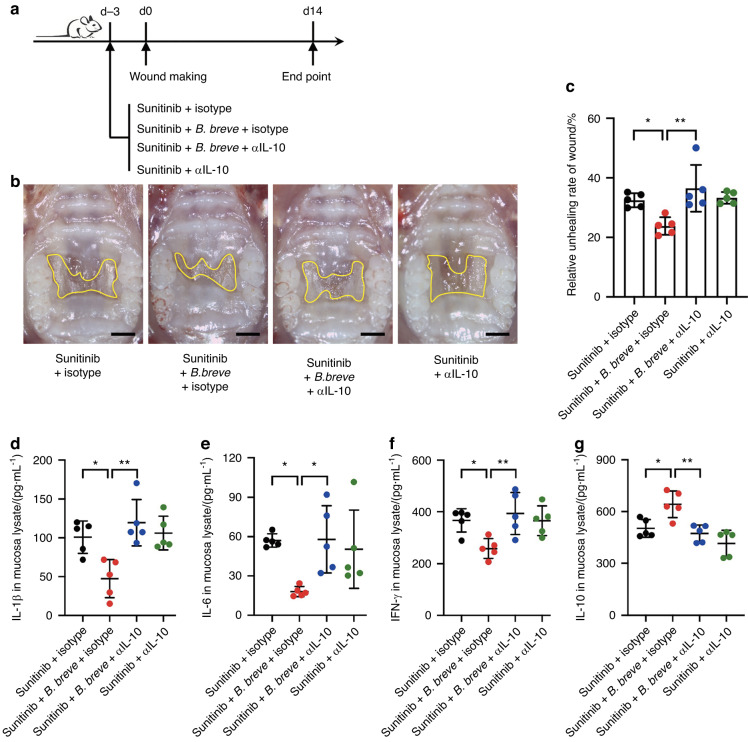
The improvement effect of *B. breve* depended on IL-10. **a** Schematic diagram of the experimental protocol. **b** Macroscopic observation of the palate mucosal wound healing in mice after different treatments (Scale bar: 1 mm). **c** Quantitative analysis of the relative wound area after treatment for 14 days. Each dot represents one mouse in two independent experiments of five mice per group. **d**–**g** The expression level of IL-1β, IL-6, IFN-γ, and IL-10 in the mucosa detected by ELISA. The data were analyzed with one-way ANOVA and presented as mean ± SD (*P < 0.05; **P < 0.01)

Finally, to verify that IL-10 was the main factor for improving the healing of oral mucosal wounds, we used a neutralizing antibody for IL-10 (αIL-10) in mice receiving *B. breve* gavage. Our results showed improved wound-healing ability of mice due to *B. breve* (Fig. 7a–c). Moreover, the effect of *B. breve* treatment upon inflammatory factors (IL-1β, IL-6, and IFN-γ) also vanished, compared with the mice that did not receive the neutralizing antibody (Fig. 7d–f). Naturally, *B. breve*-caused increase of IL-10 was neutralized by αIL-10 (Fig. 7g). These results validated that oral administration of *B. breve* promotes sunitinib-induced oral mucosal wound healing via IL-10.

## Discussion

Anti-angiogenic drugs have been widely used to treat advanced malignant tumors. In this study, we selected sunitinib, currently the most commonly used first-line drug in clinical practice, as a representative drug. Adverse reactions such as hepatotoxicity, cardiotoxicity, and hypertension caused by sunitinib are well-known issues.^26^ However, only a few studies have reported the barriers to oral mucosal healing caused by sunitinib. In this study, the mouse hard palate mucosal defect model was used to investigate the effect of sunitinib on mucosal healing, which is a valuable exploration line to advance the understanding of the adverse reactions of anti-angiogenic drugs.

The healing of oral mucosal wounds requires four successive and overlapping stages, i.e., hemostasis and coagulation, inflammation, cell proliferation, tissue remodeling and maturation.^27^ Many factors can interfere with one or more phases of the oral mucosal wound healing process, thus causing improper or impaired wound healing.^2,28^ More recently, a great deal of research has been directed at understanding the critical factors that influence poorly healing wounds.^29,30^ Following the initial hemostasis phase, the wound undergoes immediate inflammatory infiltration in response to chemokines at the site of the injury.^31^ At the same time, inflammatory cells, including neutrophils, macrophages, and lymphocytes, migrate into the wound.^2,32^ The inflammatory response influences the entire healing process, and its imbalance potentially leads to excessive tissue destruction, wound infection, and delayed wound healing.^33^

The proliferative phase generally follows and overlaps with the inflammatory phase and is characterized by epithelial proliferation and migration over the provisional matrix within the wound (re-epithelialization). Mesenchymal stem cells produce many bioactive molecules that can promote stem/progenitor cell proliferation, determine the direction of differentiation, enhance angiogenesis and even modulate immune responses, leading to wound healing and tissue repair.^34^ Many studies have reported exogenous implantation of stem cells, or stem cell secretions, to promote wound healing.^34,35^ OMSCs have been reported to be a potential solution to tackle wound healing impairment.^36^ Cellular senescence is a physiological mechanism whereby a proliferating cell undergoes a stable cell cycle arrest upon damage or stress and elicits SASP, which includes pro-inflammatory cytokines such as TNF-α, IFN-γ, IL-6, etc.^37,38^ Herein, we found that sunitinib induced cellular senescence in OMSCs, which not only interfered with their immunomodulatory function but also resulted in the secretion of pro-inflammatory factors. This process might be the mechanism of oral mucosal wound healing impairment caused by anti-angiogenic drugs.

**Fig. 8:**
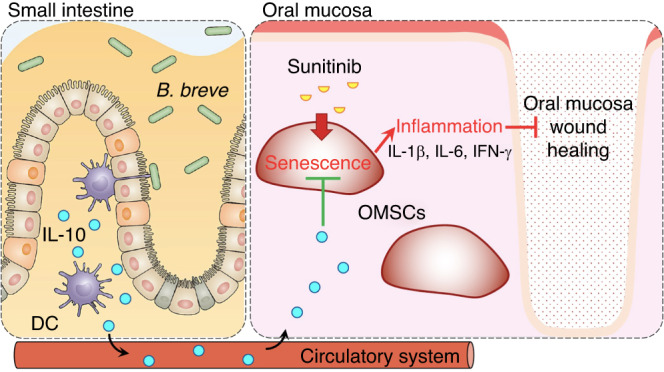
Sunitinib-induced OMSCs senescence leads to oral mucosa wound healing impairment. *B. breve* could reduce oral mucosal inflammation and promote wound healing via intestinal dendritic cells (DCs)-derived IL-10

Probiotics might become a novel therapeutic method for wound healing.^39,40^ Yet, the precise mechanism of action is still not fully understood, but a systemic influence on the immune response by balancing pro- and anti-inflammatory agents has been described.^41^ Han et al. reported that *Lactobacillus reuteri* extracts could activate the potentials of OMSCs, thus promoting wound healing via PI3K/AKT/β-catenin/TGFβ1 pathway.^42^ Previous clinical studies have suggested that beneficial bacteria may accelerate the healing of surgical wounds and burns, and diabetic foot ulcers.^40,43,44^ However, there are few reports on probiotics improving wound healing under anti-angiogenic drug treatment conditions. Our previous studies suggested that after oral administration of probiotics, the intestinal environment-immune cell interaction regulates the immune state and reduces the tumor burden.^19^ IL-10 is an anti-inflammatory cytokine that helps regulate the immune response, reinforcing skin wound healing and functional recovery after a brain or spinal cord injury.^45–48^ We found that oral administration of *B. breve* could promote intestinal DCs to secrete IL-10, reduce the inflammatory state of the oral mucosa, and improve oral mucosal healing under sunitinib treatment (Fig. 8). Notably, the neutralizing antibody for IL-10 completely counteracted the pro-healing efficacy derived from oral *B. breve* in mice.

Although we obtained some interesting findings in this study, the molecular mechanism was not fully elucidated, and further investigation is needed to reveal how sunitinib causes cellular senescence in OMSCs. Relevant studies will hopefully help us to understand anti-angiogenic drugs-derived oral mucosal wound healing impairment and MRONJ in more depth.

In conclusion, we explored the mechanisms of oral mucosal wound healing impairment caused by anti-angiogenic drugs and oral wound healing improvement following oral administration of *B. breve*. This research provided a potential solution for wound healing during anti-angiogenic drug treatment in clinic.

## Materials and methods

### Animals

C57BL/6N mice, female and 6 weeks old, were purchased from Beijing Vital River Laboratory Animal Technology Co., Ltd. All mice were maintained under specific pathogen-free conditions with a temperature of 22 ± 1 °C and a light/dark cycle of 12/12 h. All animal studies (including the mice euthanasia procedure) were done in compliance with the regulations and guidelines of Peking University institutional animal care and conducted according to the AAALAC and the IACUC guidelines (LA2022265).

### In vivo experiments

Animal testing was divided into 4 following steps: (1) investigating the effect of sunitinib and/or zoledronate on oral mucosa defect healing; (2) investigating the effect of *B. breve* on oral mucosa defect healing impairment caused by sunitinib; (3) investigating the effect of *B. breve* on a host by ligated loop experiments; (4) investigating the effect of IL-10 by in vivo blocking treatment.

### Establishing model mice

Mice were randomly divided into four groups (6 mice per group): sunitinib group, zoledronate group, zoledronate + sunitinib group, and control group. The model groups received sunitinib (S7781, Selleck Chemicals, dissolved in corn oil) orally at 40 mg·kg^−1^ of body weight every other day and/or zoledronate (SML0223, Sigma, dissolved in normal saline) intraperitoneally at 1 mg·kg^−1^ of body weight twice a week. Drugs were administered for 3 days before making the oral hard palate mucosal defect. When mice were under general anesthesia, a square mucosal defect (about 2.5 mm × 3 mm) was created by a scalpel. The boundary of the defect was from the mesial line of the bilateral maxillary first molars to the distal line of the bilateral maxillary third molars and along the palatal sides of the bilateral maxillary molars. Subsequently, drugs (sunitinib and zoledronate) were administered for another two weeks. A control group received the same amount of normal saline and corn oil.

### *B. breve* treatment

Mice were randomly divided into four groups (5 mice per group): control group, sunitinib group, sunitinib + *B. breve* group and *B. breve* group. After modeling, *B. breve* (1 × 10^9^ in 100 μL PBS) or PBS was given to mice in the experimental or control group by gavage for 2 weeks. *B.breve* lw01 was isolated by our group previously, and was cultured in MRS Broth (BD) plus Raffinose (Amresco) supplement and 0.5% L-cysteine·HCl (Sigma-Aldrich) under an anaerobic environment at 37 °C.^49^

### Ligated loop experiments

Mice were anesthetized intraperitoneally with pentobarbital sodium (80 mg·kg^−1^ body weight). Terminal ileal ligated with stitches, forming a 2 cm long loop. Then, the ligated loops were injected with *B. breve* (1 × 10^9^ in 100 μL PBS) or PBS (6 mice per group). After 45 min, intestines were removed for immunofluorescence staining and quantitative real-time polymerase chain reaction assay (qRT-PCR).

### In vivo IL-10 blocking treatment

Mice were randomly divided into four groups (5 mice per group): sunitinib + isotype group, sunitinib + *B. breve* + isotype group, sunitinib + *B.breve* + αIL-10 group and sunitinib + αIL-10 group. Two hundred μg of anti-IL-10 neutralizing antibody (JES5-2A5, BE0049, BioXCell) or isotype antibody (HRPN, BE0088, BioXCell) was injected intraperitoneally every other day after modeling.

### Wound healing evaluation

At the end of the experiment, mice were euthanized by an overdose of carbon dioxide followed by cervical dislocation, and maxillae were dissected and collected. The mucosal wound of the oral palate was observed and photographed by stereo microscopy (S8APO, Leica). The area of the original defect and the unhealing wound were measured by ImageJ software. Formula of relative wound rate was (unhealing wound area)/(original defect area)×100%.

### Micro-computed tomography (micro-CT)

The maxillary specimens were fixed in 4% paraformaldehyde (PFA) solution at 20 °C for 24 h and then scanned using micro-CT (Inveon MM Gantry-STD, Siemens). The volume and density of the bone under the mucosal defect were analyzed using Inveon Research Workplace (Siemens). Besides, images of the three-dimensional reconstruction were obtained to show the bone differences.

### mRNA sequencing and analysis

mRNA sequencing was performed by Cloud-Seq Biotech (Shanghai, China). In brief, total RNA was extracted from murine oral mucosa using TRIzol reagent (Invitrogen). The sequencing library was constructed using NEBNext® Ultra™ II Directional RNA Library Prep Kit (New England Biolabs, Inc., Massachusetts, USA). Raw data were obtained by sequencing with the Illumina NovaSeq 6000 sequencer. Cutadapt software (v1.9.3), hisat2 software (v2.0.4), HTSeq software (v0.9.1) and edgeR were successively used to complete data processing and analysis, obtaining differentially expressed genes (DEGs). Kyoto Encyclopedia of Genes and Genomes pathway gene set (c2.cp.kegg.v7.2.symbols.gmt, KEGG) was used to perform pathway analysis.

### Protein microarray analysis

Mouse protein antibody arrays (Quantibody® Mouse TH17 Array 1, Cat. # QAM-TH17-1, RayBiotech, Inc., Guangzhou, China) were provided by RayBiotech. Both positive control spots and negative control spots were on the array. The signal intensity was calculated as the fluorescence intensity minus the local background and normalized to the positive control signals. Quantitative measurement of mouse cytokines was performed according to standard curves.

### Enzyme-linked immunosorbent assay (ELISA)

Tissue lysate or culture supernatant of cells was collected. IL-1β, IL-6, IFN-γ and IL-10 levels were determined by ELISA kits (BioLegend). The absorbance (450 nm) was measured using a microplate reader (Elx808; BioTek).

### Isolation and culture of mouse OMSCs

Mouse OMSCs were isolated from the oral mucosa of C57BL/6N mice. The tissues were cut into 0.5 mm pieces and digested with 4 mg·mL^−1^ dispase II (Roche) and 2 mg·mL^−1^ collagenase I (Sigma) for 30 min at 37 °C. Cells were collected by centrifugation at 300 g for 5 min and then filtered with a 70 μm cell strainer. Cells were cultured in α-modified minimum essential medium (α-MEM, Gibco) containing 10% fetal bovine serum (ExCell), 100 IU·mL^−1^ penicillin, and 100 μg·mL^−1^ streptomycin (Gibco) in a humidified atmosphere containing 5% CO_2_/95% air at 37 °C. OMSCs at passages 2–3 were used for experiments.

### Flow cytometry

Single-cell suspension of OMSCs was prepared. The following antibodies were used: PE-CD44 (IM7), PE-CD105 (MJ7/18), PE-CD34 (MEC14.7), and respective isotype controls from BioLegend, PE-CD45 (30-F11) and isotype control from eBioscience. Analysis was performed using a flow cytometer (DxP Athena, Cytexbio).

### Senescence-associated β-galactosidase (SA-β-Gal) staining

An SA-β-Gal Staining Kit (G1580, Solarbio, Beijing, China) was employed to investigate the senescence of OMSCs after treating cells with 500, 1 000 nmol·L^−1^ sunitinib (S7781, Selleck Chemicals) or 100 ng·mL^−1^ IL-10 (575802, Biolegend). Briefly, cells cultured in a six-well plate were incubated with 1 mL of stationary liquid for 15 min at room temperature. After washing with PBS, cells were incubated with 1 mL of β-galactosidase staining working solution overnight at 37 °C. Images of the cells were taken by an inverted microscope (Olympus) and analyzed using ImageJ software.

### Immunofluorescence

Cells or cryosections of intestines were fixed in 4% PFA for 10 min at room temperature and then incubated with the primary and fluorescent secondary antibodies. The following antibodies were used: anti-γH2A.X (20E3) and anti-CD11c (D1V9Y) from CST, anti-CCL20 (ab9829) from Abcam, and goat anti-rabbit IgG H&L secondary antibodies from ZSGB-Bio. Nuclear staining was performed by incubation with 4ʹ,6-diamidino-2-phenylindole (DAPI, ZSGB-Bio). The cells or cryosections were visualized using a fluorescent optical microscope (Olympus). Five representative fields were randomly selected for statistical analysis.

### Cell proliferation assay

OMSCs were plated at a density of 2 000 cells per well in a 96-well plate and treated with 500, 1 000 nmol·L^−1^ sunitinib (S7781, Selleck Chemicals) or 100 ng·mL^−1^ IL-10 (575802, Biolegend) for 1, 3, 5 and 7 days. At each time point, 10 μL cell counting kit-8 (CCK-8) solution was added to each well and incubated for 2 h at 37 °C. The absorbance (450 nm) was measured using a microplate reader (Elx808; BioTek).

### Wound healing assay

OMSCs were cultured in a 6-well plate. After incubation in serum-free medium for 24 h, a line was drawn using a marker on the bottom of the dish, and then a sterile 200-μL pipet tip was used to scratch three separate wounds through the cells, moving perpendicular to the line. The cells were gently rinsed twice with PBS to remove floating cells and incubated with 500, 1 000 nmol·L^−1^ sunitinib (S7781, Selleck Chemicals) or 100 ng·mL^−1^ IL-10 (575802, Biolegend) in serum-free medium. Images of the scratches were taken by an inverted microscope (Olympus) at 0 h and 24 h after sunitinib or IL-10 treatment and analyzed using ImageJ software.

### Alizarin red staining

After culturing with an osteogenic differentiation medium for 14 days, OMSCs were stained with alizarin red according to the manufacturer’s protocol (Oricell). Images of the cells were taken by an inverted microscope (Olympus).

### Oil Red O staining

After culturing with adipogenic differentiation medium for 14 days, OMSCs were stained by Oil Red O according to the manufacturer’s protocol (Oricell). Images of the cells were taken by an inverted microscope (Olympus).

### Quantitative real-time polymerase chain reaction assay (qRT-PCR)

Total RNA was extracted from tissues or cells by TRIzol. Quantification of mRNA expression was performed using FastStart Universal SYBR Green Master (ROX) reagent (Roche) on an ABI Step One Plus Real-Time PCR System. β-actin was used as the endogenous standard. The PCR program consisted of predenaturation at 95 °C for 10 min followed by 40 cycles amplification of 95 °C for 15 s and 60 °C for 1 min. Sequences of the primers are shown in Supplementary Table 1.

### Generation of bone-marrow-derived dendritic cells (BMDCs)

Bone marrow cells were isolated from femurs and tibiae of C57BL/6 N mice and then cultured in Roswell Park Memorial Institute (RPMI)-1640 medium (Gibco) containing 10% fetal bovine serum (ExcCell), 100 IU·mL^−1^ of Penicillin and 100 ng·mL^−1^ of Streptomycin (Gibco), 25 ng·mL^−1^ rmGM-CSF and 12.5 ng·mL^−1^ rmIL-4 (BioLegend) for 8 days. BMDCs were then stimulated for 4 or 24 h without or with *B. breve* at a ratio of 1:100.

### Statistical analysis

The statistical analysis of the cell-proliferation curve was performed by GraphPad Prism 7 with two-way ANOVA. The other statistical analyses were performed with one-way ANOVA or two-tailed Student’s *t* test. *P* < 0.05 represented statistical significance.

### Supplementary information

Supplementary Figures

Supplementary Table 1

Raw data of mRNA sequencing

Raw data of protein microarray analysis

## Data Availability

All data generated or analyzed during this study are included in this published article and its supplementary information files.
