# Supplementary material for: Oral administration of Bifidobacterium breve improves anti-angiogenic drugs-derived oral mucosal wound healing impairment via upregulation of interleukin-10
Source: Int J Oral Sci. 2023 Dec 11;15:56. doi: 10.1038/s41368-023-00263-y (PMC10711028; doi:10.1038/s41368-023-00263-y)
Supplement: Supplementary file 1 — Supplementary Figures [file 41368_2023_263_MOESM1_ESM.docx]

**Supplementary Figures**


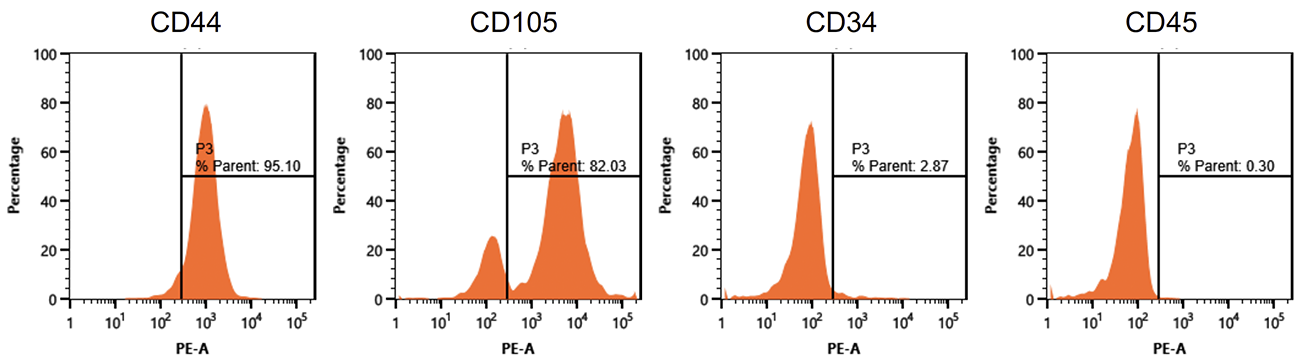


Fig. S1. **Identification of OMSCs.** Flow cytometric analysis of OMSCs for mesenchymal stem cell surface markers CD44 and CD105, and the hematological markers CD34 and CD45.


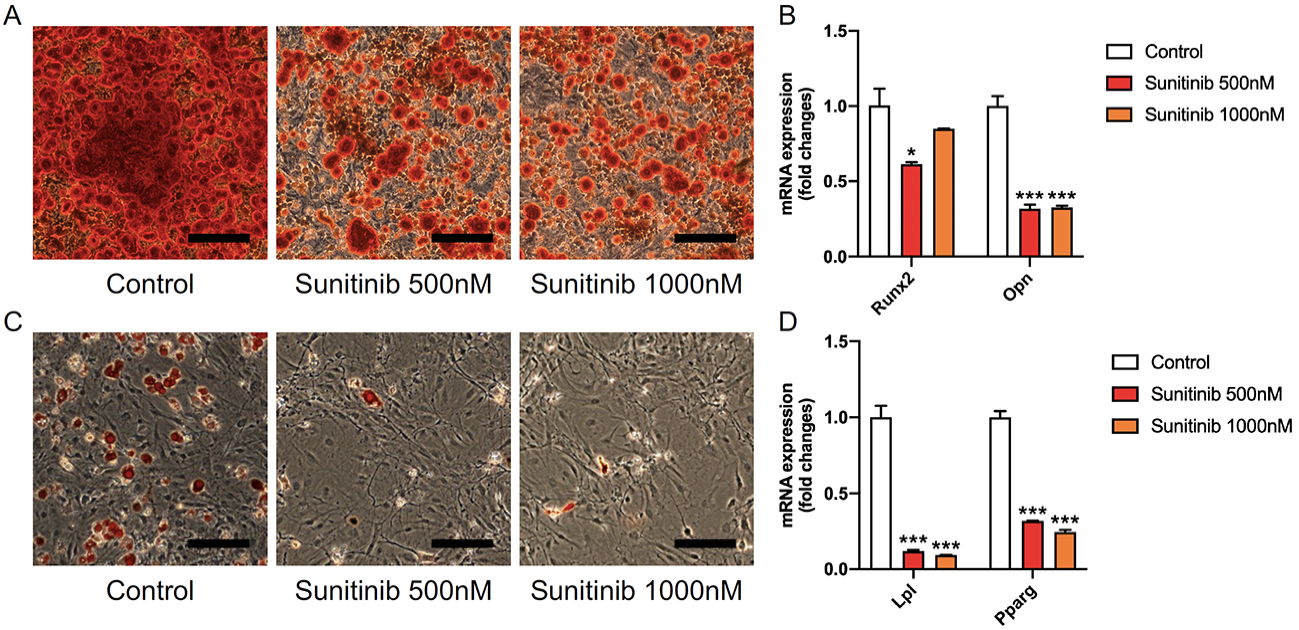


Fig. S2. **Anti-angiogenic drug sunitinib inhibited the differentiation capacity of OMSCs.** **(A)** Alizarin red staining (scale bars: 50 μm). **(B)** The expression of the crucial transcription factors for modulating osteogenic differentiation (Runx2 and Opn) detected by qRT-PCR. **(C)** Oil Red O staining (scale bars: 50 μm). **(D)** The expression of the crucial transcription factors for modulating adipogenic differentiation (Lpl and Pparg) were detected by qRT-PCR. Actin was used as an internal control. The data were analyzed with one-way ANOVA and presented as mean ± SD (**P* < .05; ****P* < .001).


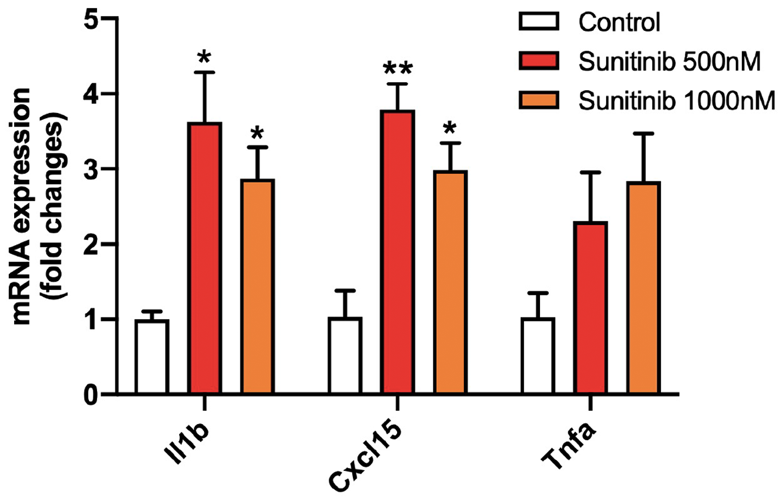


Fig. S3. **Anti-angiogenic drug sunitinib promoted the expression of inflammatory factors in OMSCs.** The expressions of major inflammatory factors were detected by qRT-PCR. Actin was used as an internal control. The data were analyzed with one-way ANOVA and presented as mean ± SD (**P* < .05; ***P* < .01).
